# The Effects of Different Theta and Beta Neurofeedback Training Protocols on Cognitive Control in ADHD

**DOI:** 10.1007/s41465-022-00255-6

**Published:** 2022-11-04

**Authors:** Annet Bluschke, Elena Eggert, Julia Friedrich, Roula Jamous, Astrid Prochnow, Charlotte Pscherer, Marie Luise Schreiter, Benjamin Teufert, Veit Roessner, Christian Beste

**Affiliations:** Cognitive Neurophysiology, Department of Child and Adolescent Psychiatry, Faculty of Medicine, TU Dresden, Schubertstrasse 42, 01309 Dresden, Germany

**Keywords:** ADHD, Neurofeedback, Cognitive control, Executive functions, Theta oscillations, Beta oscillations

## Abstract

Neurofeedback (NF) is an important treatment for attention deficit/hyperactivity disorder (ADHD). In ADHD, cognitive control deficits pose considerable problems to patients. However, NF protocols are not yet optimized to enhance cognitive control alongside with clinical symptoms, partly because they are not driven by basic cognitive neuroscience. In this study, we evaluated different EEG theta and/or beta frequency band NF protocols designed to enhance cognitive control. Participants were *n* = 157 children and adolescents, *n* = 129 of them were patients with ADHD (*n* = 28 typically developing (TD) controls). Patients with ADHD were divided into five groups in the order of referral, with four of them taking part in different NF protocols systematically varying theta and beta power. The fifth ADHD group and the TD group did not undergo NF. All NF protocols resulted in reductions of ADHD symptoms. Importantly, only when beta frequencies were enhanced during NF (without any theta regulation or in combination with theta upregulation), consistent enhancing effects in both response inhibition and conflict control were achieved. The theta/beta NF protocol most widely used in clinical settings revealed comparatively limited effects. Enhancements in beta band activity are key when aiming to improve cognitive control functions in ADHD. This calls for a change in the use of theta/beta NF protocols and shows that protocols differing from the current clinical standard are effective in enhancing important facets of cognitive control in ADHD. Further studies need to examine regulation data within the neurofeedback sessions to provide more information about the mechanisms underlying the observed effects.

**Supplementary Information:**

The online version contains supplementary material available at 10.1007/s41465-022-00255-6.

## Introduction

Attention deficit hyperactivity disorder (ADHD) is a prevalent, multi-faceted disorder in childhood and adolescence. Apart from pharmacological treatments using stimulants (e.g. methylphenidate) or non-stimulants (e.g. atomoxetine), non-pharmacological treatment approaches are increasingly applied (dosReis et al., 2017; Ng et al., 2017; Schatz et al., 2015). One major approach is “neurofeedback training” (NF) which has frequently been shown to ameliorate ADHD symptoms and associated problems (Arns & Strehl, 2013; Arns et al., 2009; Bluschke et al., 2016a, 2016b; Gevensleben et al., 2010; Holtmann et al., 2014; Lofthouse et al., 2012; Micoulaud-Franchi et al., 2015). The basic principle behind NF is that EEG-recorded brain activity is visualized and directly fed back to patients, e.g. via simple computer games. Using this feedback, the patient trains to up- or downregulate a predefined aspect of brain activity that is thought to be associated with the desired behavioural outcomes (like increased attention in the case of ADHD, for example). Building on this association, NF is thought to exert therapeutic effects based on the principles of operant conditioning and increasing habituation. There are several forms of NF, the most established form of which (“theta/beta NF”) could be called the “gold standard” of NF. During this approach, the commonly applied procedure in clinical settings is to train the patient to decrease activity in the theta (θ) frequency band and to concomitantly increase activity in the beta (β) frequency band (i.e. a θ↓β↑ protocol). This protocol has been developed on the basis of two considerations: theta frequency activity has commonly been interpreted as an indicator of daydream-like, in-alert states (i.e. this state should be decreased in ADHD), while beta band activity has been thought to reflect a state of focused attention and mental arousal (i.e. this state should be increased in ADHD) (Bluschke et al., 2016a, 2016b).

Although meta-analyses and review articles corroborated that this form of NF ameliorates clinically assessed ADHD symptoms (Enriquez-Geppert et al., 2019; Lambez et al., 2020; Lofthouse et al., 2012; Riesco-Matías et al., 2021; Van Doren et al., 2019; Yan et al., 2019), this procedure is not without problems (Bluschke et al., 2016a, 2016b; Catalá-López et al., 2017; Cortese et al., 2016; Razoki, 2018). Rather than solely relying on rather subjective symptom assessments, it seems useful to employ objective measures of functionality when evaluating the effects of neurofeedback (Berger, 2011; Emser et al., 2018). In the case of ADHD, such objective measures commonly are measures of different domains of executive functions resp. cognitive control (Diamond, 2013) which, albeit not universally deficient in all affected patients (Willcutt et al., 2005), are frequently reported and represent a clinically relevant facet of the disorder (Faedda et al., 2019; Krieger & Amador-Campos, 2018; Krieger et al., 2019; Miklós et al., 2019; Skogli et al., 2017), especially as far as academic functioning (Sibley et al., 2019) and time spent on task (Dekkers et al., 2017) are concerned. Importantly, it has been argued (Bluschke et al., 2016a, 2016b) that the currently widely established θ↓β↑ NF protocol may not be optimal when aiming to ameliorate difficulties in executive functions in ADHD. Especially, it does not seem useful to train patients with ADHD to downregulate theta band activity (as done in the standard θ↓β↑ NF protocol) when aiming to improve cognitive control deficits (e.g. response inhibition and interference control). This presumption is based on the fact that several lines of basic cognitive neuroscience research have shown for high performance in inhibitory control and (attentional) interference control to be associated with a transient upregulation of processes for which theta band activity is relevant (Cavanagh & Frank, 2014; Cohen, 2014; Wascher et al., 2012). From the basic cognitive neuroscience point of view, a training protocol focussing on the increase of theta band activity thus seems more suitable (Bluschke et al., 2016a, 2016b). Concerning beta band activity, its functional relevance and importance in theta/beta NF protocols and its effect on cognitive control processes in ADHD is largely elusive. Basic research suggests that beta band activity is important to allow a high functioning of cognitive (attentional) and sensorimotor systems (Engel & Fries, 2010; Studer et al., 2014; Womelsdorf & Everling, 2015) and enables response regulation processes (Bluschke et al., 2020; Dahan et al., 2018) allowing sensory information to efficiently guide subsequent motor actions (MacKay, 1997). This may be the reason why training to upregulate beta band activity is useful for the enhancement of cognitive control processes (Bluschke et al., 2020, 2016a, 2016b). Concerning ADHD, some data suggest that particularly the modulation of beta band activity may be of importance (Bluschke et al., 2020).

No study has yet presented a systematic comparison of NF protocols modulating theta and/or beta frequencies. Specifically, a comparison against the θ↓β↑ protocol reflecting the “gold standard” of NF concerning the effectiveness in modulating different cognitive control functions seems necessary. The current study closes this gap. Specifically, it aims to examine the effects of theoretically driven manipulations of the classical NF protocol on objective performance measures assessing cognitive control functions bearing a significant relevance for the day-to-day functioning of patients with ADHD. As primary outcome measures, we thus focus on response inhibition/impulsivity examined by a Go/Nogo task and on (attentional) interference control examined by a flanker task (see “Methods” section for details). We compare various ADHD groups receiving different NF protocols: (i) a group receiving a standard θ↓β↑ NF protocol (θ↓β↑), (ii) a group receiving a NF protocol to upregulate both theta and beta frequency activity (θ↑β↑), (iii) a group receiving a NF protocol to only upregulate theta band activity (θ↑) and (iv) a group receiving a NF protocol to only upregulate beta band activity (β↑). Aside from the direction of modulation of theta and/or beta band activity, the NF procedure was identical in all groups (see “Methods” section for details). Since basic neuroscience research suggests that cognitive control requires enhanced theta and beta oscillations (Bluschke et al., 2020, 2016a, 2016b), this study did not include protocols which only aimed at training participants to decrease power in both or one of these frequency bands. Before and after NF, the same Go/Nogo and flanker tasks were used to test response inhibition and interference control performance. In addition to the ADHD groups treated with NF, a group of patients with ADHD not taking part in NF was examined to control for retest effects (no NF). Similarly, a group of typically developing healthy controls (TD) not receiving an intervention was examined.

We hypothesize that changes in task performance before vs. after NF training (in the Go/Nogo and the flanker task) will be stronger in NF protocols training the upregulation of theta and/or beta frequency bands than it is the case for the standard θ↓β↑ NF protocol. Concerning the Go/Nogo task, we hypothesize that specific effects will occur in inhibition errors (i.e. Nogo false alarms), since demands on cognitive control depending on theta/beta oscillations are particularly high in these trials. For similar reasons, we expect specific effects in the flanker task due to modulations in the incompatible condition (i.e. where flanker and target stimuli point into opposite directions). We hypothesize that patients with ADHD and typically developing controls not receiving an intervention between testing time points will not show any changes in performance in the Go/Nogo and the flanker task. At present, no clear-cut hypotheses can be stated as to whether stronger or more consistent effects of the examined NF protocols will be evident for the different examined cognitive control functions. Further, it is important to stress that our design has not been conceptualized in order to compare the effects of different NF protocols on clinical parameters or even their effectiveness. Instead, the goal was to examine the effects of theoretically driven manipulations of the common NF protocol on objective performance measures by assessing cognitive control functions which bear a significant relevance for the day-to-day functioning of patients with ADHD. To provide a basic estimate of symptom severity and changes within it, we additionally report parent-rated ADHD symptoms as measured using the AD(H)D Symptom Checklist (Döpfner et al., 2008) (secondary outcome measure). Further, it will also be examined whether any changes in the power of the frequency bands at rest (i.e. during the baseline measurements) and during training (i.e. “training success”) actually occur between the first and the last neurofeedback session.

## Methods

This study was conducted in a naturalistic setting in the outpatient department of the Clinic for Child and Adolescent Psychiatry and Psychotherapy of a large university hospital. Overall, *n* = 231 children and adolescents were included in the study between April 2014 and October 2020.

### A Priori Sample Size Estimation

In previous studies on the effects of NF in ADHD, we reported effect sizes of the treatment of approx. *η*_*p*_^2^ = 0.1 (Bluschke et al., 2018, 2016a, 2016b). This estimate was used to calculate the required sample size in the current study to detect an effect of NF treatment with a power of at least 95% (alpha level = 5%, 6 groups, 2 measurement time points, conservative estimation of the correlation among the repeated measures of *r* = 0.2). The power calculation using G*Power revealed a total necessary sample size of *n* = 84; i.e. *n* = 14 patients per group. To ensure an even higher robustness of the effects, we nearly doubled the sample size (*n* = 157) and no included group was smaller than *n* = 21 patients (Table 1).

**Table 1:** Participant flow. Table showing number of participants excluded due to missing T2 data and those excluded to achieve group matching in age, IQ and medication status

	Patients starting intervention and controls	Not analysed since no T2 data available	Excluded to achieve group matching	Final sample
TD	*n* = 30	*n* = 2	*n* = 0	*n* = 28
No NF	*n* = 32	*n* = 6	*n* = 3	*n* = 23
θ↓β↑	*n* = 66	*n* = 9	*n* = 28*	*n* = 29
θ↑β↑	*n* = 33	*n* = 4	*n* = 1	*n* = 28
θ↑	*n* = 35	*n* = 11**	*n* = 3	*n* = 21
β↑	*n* = 35	*n* = 6**	*n* = 1	*n* = 28
Total	*n* = 231	*n* = 38	*n* = 36	*n* = 157

^*^Random exclusion of participants to achieve comparable group size

^**^Due to the COVID-19 pandemic, a slightly larger proportion of participants from θ↑ and β↑ groups did not complete their training and/or the T2 assessment

### Sample Description and Study Design

**Fig. 1:**
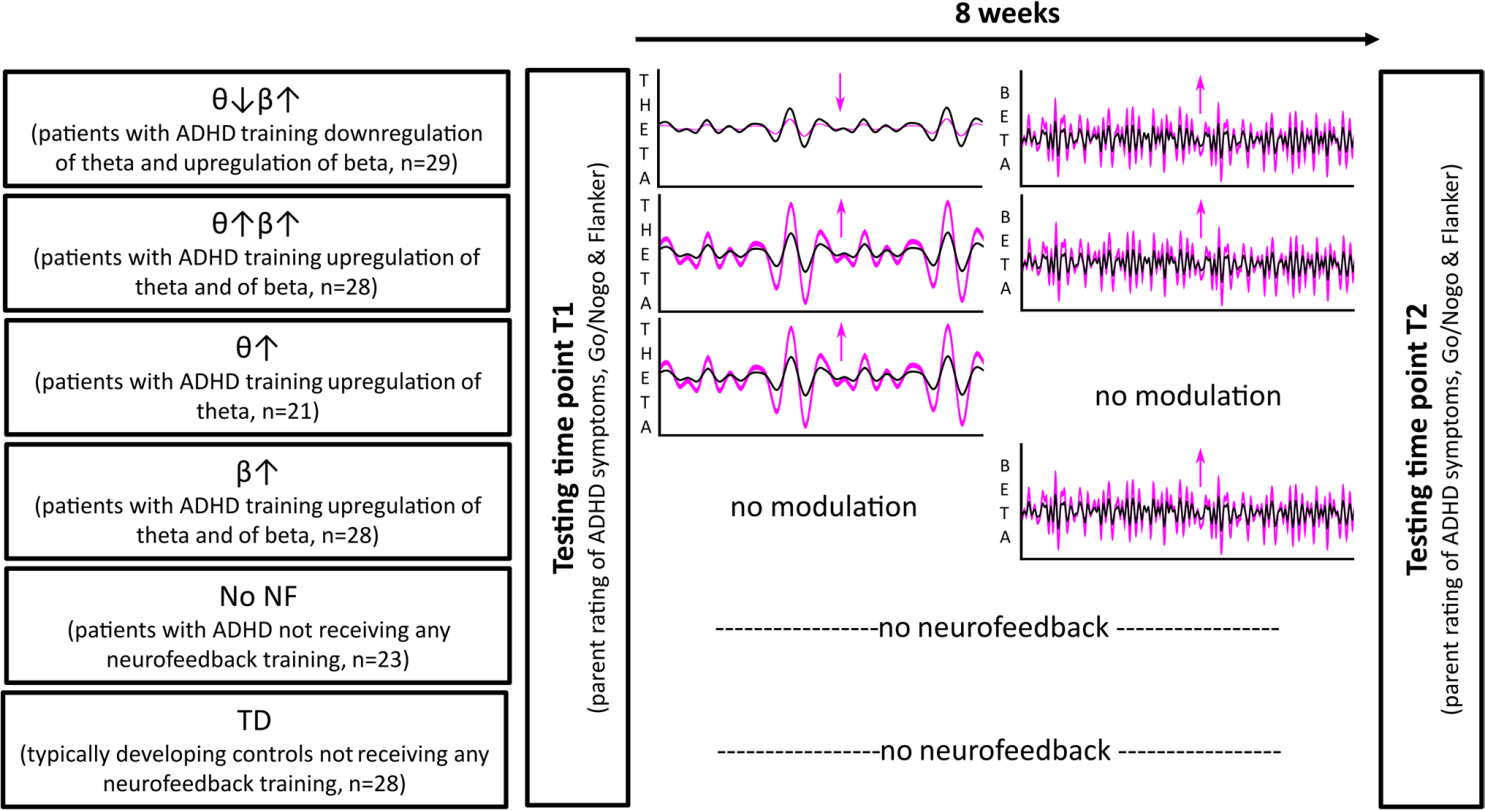
Study design. Six participant groups were included (four active neurofeedback treatment groups, two control groups). All participants were tested twice (T1 and T2) 8 weeks apart

*N* = 38 of the initially included participants did not complete their participation and were thus not included in further analyses (see Table 1). Of those who completed participation, *n* = 28 were typically developing controls who were recruited via advertisements and an in house data base. The other *n* = 169 participants were children and adolescents with ADHD, in whom ADHD diagnoses had been determined according to standard clinical guidelines by a team of experienced child and adolescent psychiatrists and psychologists (incl. family and school interviews and questionnaires, IQ and attention testing, exclusion of possible somatic differential diagnoses via blood analyses, EEG, audiometry and vision testing). To match the characteristics of the groups in regard to age, IQ, medication status and group size, some participants were subsequently excluded from analysis (see Table 1). This was done purely based on demographic data and irrespective of any outcome measures. In total, *n* = 129 patients with ADHD were eventually included in the analyses. All participants had been diagnosed in the outpatient clinic and fulfilled criteria for ADHD according to ICD-10 criteria. *N* = 14 had an axis I comorbidity (*n* = 1 OCD, *n* = 2 adjustment disorder, *n* = 6 tic disorder, *n* = 3 CD/ODD, *n* = 2 emotional problems). *N* = 22 of the ADHD patients had received an additional axis II diagnosis. Patients were only considered for participation if ADHD was the main diagnosis and if the clinical indication for neurofeedback was confirmed by the treating clinicians. The *n* = 129 patients eventually included in analyses belonged to five groups (for details, see Fig. 1/Table 2). Group allocation to the different neurofeedback groups took place in the order of referral (order: no NF, θ↓β↑, θ↑β↑, θ↑ and β↑). All participants took part in two assessments (T1 and T2) in the space of 8 weeks. These took place independent of the NF sessions and were conducted by different members of staff who were unaware of any contents of the NF training. Due to the COVID-19 pandemic, a slightly larger number of participants from θ↑ and β↑ groups did not complete their training and/or the T2 assessment (Table 1).

**Table 2:** Demographic data of all study groups. Demographic details (mean ± SE) for all six experimental groups (θ↓β↑ (patients with ADHD training downregulation of theta and upregulation of beta), θ↑β↑ (patients with ADHD training upregulation of theta and upregulation of beta), θ↑ (patients with ADHD training upregulation of theta), β↑ (patients with ADHD training upregulation of beta), no NF (patients with ADHD not receiving any neurofeedback training) and TD (typically developing healthy controls not receiving any neurofeedback training)) showing age (years), IQ points and details of medication intake (*MPH* methylphenidate, *ATX* atomoxetine, *LDEX* lisdexamfetamine)

	Age	IQ	Medication		
			Medication status (yes/no)	Type of medication	Dose of MPH
TD *n* = 28	11.3 ± 0.41	108 ± 2.3	0/28	–	–
No NF *n* = 23	11.2 ± 0.42	105 ± 3.6	14/9	MPH *n* = 12, ATX *n* = 1, LDEX *n* = 1	21.7 ± 2.5 mg (range: 10–40 mg)
θ↓β↑ *n* = 29	11.1 ± 0.32	100 ± 2.1	17/12	MPH *n* = 16, ATX *n* = 1, LDEX *n* = 1	21.7 ± 2.4 mg (range: 7.5–40 mg)
θ↑β↑ *n* = 28	11.2 ± 0.41	101 ± 2.4	11/17	MPH *n* = 10, ATX *n* = 1, LDEX *n* = 0	31.3 ± 3.5 mg (range: 20–40 mg)
θ↑ *n* = 21	10.1 ± 0.38	100 ± 5.2	9/11	MPH *n* = 8, ATX *n* = 0, LDEX *n* = 1	20.6 ± 2.2 mg (range: 10–30 mg)
β↑ *n* = 28	10.2 ± 0.30	103 ± 3.1	13/15	MPH *n* = 11, ATX *n* = 0, LDEX *n* = 1	25.0 ± 1.9 mg (range: 20–30 mg)

Participants included in four of these groups took part in different forms of NF (details see below and Fig. 1). One ADHD group was trained to downregulate theta and upregulate beta power (i.e. ratio training) (θ↓β↑). Another ADHD group was trained to upregulate both theta and beta power (θ↑β↑). Regulation was rewarded when either theta or beta power was increased successfully. A third ADHD group trained to upregulate theta only (θ↑), while a fourth ADHD group only trained beta upregulation (β↑). The fifth group of patients with ADHD did not take part in any training (no NF). Further, we included a group of typically developing control children (TD). A semi-structured telephone interview was used to confirm the absence of any other neurological or psychiatric conditions. The absence of ADHD symptoms in the TD group was confirmed using the AD(H)D Symptom Checklist (Döpfner et al., 2008) (details below). This instrument assesses ADHD criteria in the domains of inattention, hyperactivity and impulsivity. Its psychometric properties are comparable to those of the internationally commonly used Conners rating scale (Erhart et al., 2008). All participants took part in two testings (Fig. 1). The T1 testing took place before the start of NF in the four treated groups. The T2 testing took place after 8 weeks of NF. In the no NF group and the TD group, T1 and T2 also took place 8 weeks apart. During this time, no intervention took place in these groups. Any ongoing treatment for ADHD in any of the participants remained constant and no new interventions were initiated. At both time points, participants completed two computer tasks measuring varying theta-/beta-related aspects of executive functions. The Go/Nogo task was used to assess behavioural inhibition while the flanker task measured conflict processing (details see below). After each of these appointments, participant families received 5 Euros as compensation. Parents were asked to complete the ADHD Symptom Checklist (Döpfner et al., 2008) at both time points. All groups do not differ from each other in terms of age (*F*(5,149) = 2.01; *p* = 0.08; *η*_*p*_^2^ = 0.06) or IQ (*F*(5,142) = 1.28; *p* = 0.28; *η*_*p*_^2^ = 0.05) as assessed by the WISC-IV or V (Table 2). There were no differences between the five included ADHD groups regarding medication status (χ^2^(4) = 3.6, *p* = 0.47) (Table 2). Medication intake was kept stable across the duration of study participation in all groups. Both laboratory testings were conducted in the afternoons to ensure comparability between the T1 and T2 appointment.

Using the AD(H)D Symptom Checklist (Döpfner et al., 2008), parents rated their children on a scale of 0 (no problems) to 3 (severe problems) in regard to the ADHD core symptoms of inattention, hyperactivity and impulsivity. Due to compliance problems, complete questionnaire data (i.e. both at T1 and T2) were only available for *n* = 99 subjects. ADHD symptom levels at T1 or T2 did not differ significantly between the five ADHD groups (see below). Typically developing controls presented with significantly lower ratings than patients with ADHD in all domains (all *t* ≥ 5.3; all *p* ≤ 0.001) (see Table 3 for details and descriptive data).

**Table 3:** Parent-rated ADHD symptoms. Parent-rated ADHD symptoms (mean ± SE) for all six experimental groups (θ↓β↑ (patients with ADHD training downregulation of theta and upregulation of beta), θ↑β↑ (patients with ADHD training upregulation of theta and upregulation of beta), θ↑ (patients with ADHD training upregulation of theta), β↑ (patients with ADHD training upregulation of beta), no NF (patients with ADHD not receiving any neurofeedback training) and TD (typically developing controls not receiving any neurofeedback training)) showing scores in the domains of inattention, hyperactivity and impulsivity. Clinical cut-offs: ≥ 1.0: clinically relevant; ≥ 1.5: severe symptoms. Due to compliance issues, only *n* = 99 complete questionnaire data sets are available

	Inattention		Hyperactivity		Impulsivity	
	T1	T2	T1	T2	T1	T2
TD *n* = 25 complete datasets	0.5 ± 0.09	0.4 ± 0.09	0.1 ± 0.06	0.1 ± 0.05	0.3 ± 0.11	0.3 ± 0.11
no NF *n* = 11 complete datasets	2.0 ± 0.26	1.8 ± 0.23	1.2 ± 0.26	1.0 ± 0.21	1.6 ± 0.30	1.4 ± 0.23
θ↓β↑ *n* = 21 complete datasets	1.9 ± 0.13	1.7 ± 0.13	1.2 ± 0.15	1.0 ± 0.15	1.6 ± 0.14	1.5 ± 0.14
θ↑β↑ *n* = 19 complete datasets	2.1 ± 0.11	1.6 ± 0.13	1.2 ± 0.18	0.9 ± 0.16	1.6 ± 0.18	1.2 ± 0.19
θ↑ *n* = 11 complete datasets	1.7 ± 0.16	1.5 ± 0.21	0.8 ± 0.20	0.7 ± 0.19	1.5 ± 0.28	1.0 ± 0.22
β↑ *n* = 12 complete datasets	1.9 ± 0.10	1.7 ± 0.13	0.9 ± 0.13	0.7 ± 0.19	1.5 ± 0.20	1.1 ± 0.21

All participants and their parents or legal guardians provided written informed consent before any study procedure was commenced. The study was conducted according to the Declaration of Helsinki and approved by the ethics committee of the Medical Faculty of the TU Dresden.

### Neurofeedback Protocols and Training Procedures

The different NF protocols and study groups are schematically illustrated in Fig. 1.

For all groups, NF training took place in two weekly sessions (1 h each) over a period of 8 weeks. NF was administered by a small team of trained psychologists in the outpatient clinic. To the participating children and adolescents, neurofeedback was introduced as an “attention training on the computer” during which they were supposed to make a cartoon character/race car move on the screen. Apart from pointing out the relevance of attention in general, no instructions on how to achieve this were provided. Participants’ strategies were to be collected during the sessions in order to increase transferability to day-to-day life. Apart from the differences in the trained frequency band(s), all procedures and elements of the treatment were kept constant across participants. The EEG in the theta (4–8 Hz) and/or beta frequency range (13–20 Hz) were recorded at electrode Cz. Eye movements were recorded from electrodes above and below the left eye. These recordings were used to correct for motion artefacts online since these could alter the results if not accounted for. The reference electrode was placed on the left mastoid and an electrode on the forehead was used as the ground electrode. Theta and beta frequency ranges were shown to participants via a custom-made software (“Self-regulation and Attention Management” (“SAM”), University of Erlangen). Time intervals containing artefacts occurring as a result of excessive movement were discarded online and were not included in the feedback procedure. Strong movement artefacts, as well as blinking artefacts, were removed online since they may affect the estimation of theta activity (max 100 µV). In case of excessive movements, children were presented with a sad smiley face in order to remind them to reduce movements. This was also shown in case of activity below a lower threshold (5 µV), in which case the connection of the electrode to the skull was checked and re-established by the psychologist if needed. For artefacts in the frequency range of 25–35 Hz, the bound was defined at 10 μV. Theta and beta power at rest was recorded in a 2-min interval at the beginning of each session (baseline). This data was analysed in order to examine whether any changes had occurred as a result of the training (see “Neurofeedback Baseline and Training Data” section for details). Unfortunately, due to technical issues (one irreparable laptop used for neurofeedback training and data recording), baseline and training data is only available for *n* = 73 of the *n* = 106 participants who had taken part in the neurofeedback training. During training, children were tasked with trying to move a cartoon character on the screen by regulating the frequencies as required (immediate and continuous feedback). Within the animation, frequency changes were shown to participants via moving bars on the screen. In each session, three to six NF blocks were conducted (5–10-min duration).

From neurofeedback session 4 or 5 onwards, transfer blocks were introduced (delayed feedback). Here, children were required to perform a task (e.g. attention game, reading, schoolwork) without being given direct feedback. Instead, they received delayed feedback showing them in how far they were able to regulate the theta and/or beta power in the required direction. Performance was reviewed with the participant at the end of each block (with immediate or delayed feedback). To ensure comparability with standard protocols (Gevensleben et al., 2010, 2009a, 2009b), NF additionally contained elements of behavioural therapy (e.g. psychoeducation, homework, token system). Strategies on how to achieve control over the feedback animation were not provided to participants. However, during the course of training, participants were asked about any strategies they employed during neurofeedback. As part of the homework, participants were tasked with applying these individual strategies (e.g. “focus on one thing only”) in attention-demanding situations in school or at home.

Due to the nature of the experimental variation (i.e. group allocation in order of referral), it was not feasible to conduct the neurofeedback training in a blinded fashion. However, staff conducting testings at time point T1 and T2 and analysing behavioural data were unaware of group membership. In addition, although participants had been informed about the use of different neurofeedback protocols within the study, this was of no practical implication to them during training since they were simply instructed as to which frequency bands needed to be regulated in which direction.

### Tasks

During the Go/Nogo task (Beste et al., 2011; Chmielewski et al., 2015), either the word “DRÜCK” (German for “PRESS”; Go stimulus) or “STOP” (Nogo stimulus) was presented for 300 ms in white font on a black background. Participants were required to perform a button press with the right index finger as fast as possible (i.e. within 500 ms) after seeing the Go stimulus. In contrast, when seeing the Nogo stimulus, participants were asked to refrain from responding. The inter-trial interval (ITI) was jittered between 1600 and 1800 ms. In total, the experiment consisted of 248 Go trials and 112 Nogo trials presented in four blocks a pseudo-randomized order. The task lasted approximately 20 min. For the analysis, we examined the rate of Nogo false alarms, the rate of correct Go hits as well as reaction times in the Go trials.

In the flanker task, vertically arranged white arrowheads were presented in the middle of the screen on a black background. Target stimuli pointing to the left or right were displayed in the centre of the screen. Two hundred milliseconds before target onset, two arrowheads (i.e. flanker stimuli) were presented above and below the target. These flanker stimuli were pointing in the same (compatible, 67% of trials) or opposite (incompatible, 33% of trials) direction as the target arrow. Target stimuli were presented for 300 ms. Flanker stimuli were displayed until target onset. The response-stimulus interval was pseudo-randomized between 1400 and 1800 ms. In order to enhance the conflict, time pressure was administered by asking participants to respond within 450 ms after target onset. A warning tone (1000 Hz, 60 dB SPL) was presented 1200 ms after the response if it had occurred more than 450 ms after the target. The flanker task consisted of four blocks of 120 stimuli each (i.e. 480 trials in total) and lasted approximately 20 min. For the analysis, we examined the reaction times and error rates in compatible and incompatible trials. Due to a technical error, T2 flanker data is missing for one participant in the θ↓β↑ group and the θ↑β↑ group.

## Results and Statistical Analyses

### Statistical Analysis

Mixed-effects ANOVAs including the within-subject factor *Time Point* (T1 vs. T2) and the between-subjects factor *Group* (θ↓β↑ vs. θ↑β↑ vs. θ↑ vs. β↑ vs. no NF vs. TD) were used to examine the effects of the four NF protocols on task performance. Analyses in the flanker task additionally included the within-subject factor *Compatibility* (compatible vs. incompatible trials). Any significant interactions were followed up by ANOVAs and/or independent/paired-sample *t*-tests. Greenhouse–Geisser correction was applied and post hoc tests were Bonferroni-corrected as necessary.

### Go/Nogo Task

**Fig. 2:**
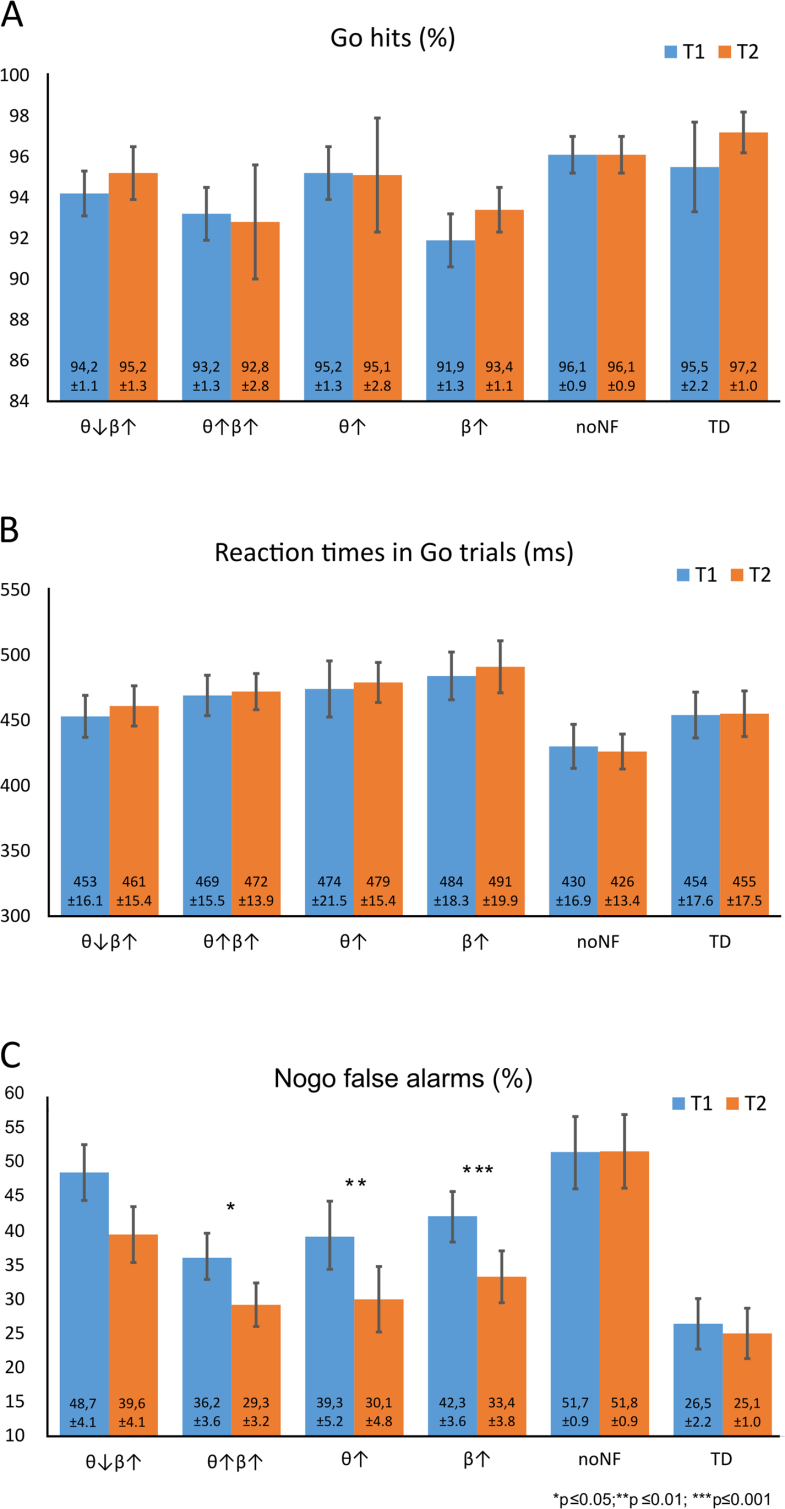
Descriptive statistics (mean ± SE) for Go hits (**A**), Go reaction times (**B**) and Nogo false alarms (**C**). Blue bars represent testing time point T1; orange bars show testing time point T2. Data is shown for four groups of patients with ADHD treated with four different neurofeedback protocols (θ↓β↑: trained to downregulate theta and upregulate beta power, θ↑β↑: trained to upregulate both theta and beta power, θ↑: trained to upregulate theta only, β↑: trained to upregulate beta only) and for two control groups (no NF: patients with ADHD who did not take part in any training, TD: typically developing control children). Asterisks indicate level of significance of conducted post hoc *t*-tests

Behavioural parameters showing performance in the Go/Nogo task are shown in Fig. 2.

Data concerning the Go trials (accuracy and reaction times) revealed no effects and is presented in the supplemental material. Descriptive data are shown in Fig. 2A and B.

Concerning Nogo false alarms, we found a significant main effect of *Time* (*F*(1,149) = 29.8; *p* ≤ 0.001; *η*_*p*_^2^ = 0.17; T1: 40.8 ± 1.7%; T2: 34.9 ± 1.7%), indicating a general performance improvement at time point T2. In addition, we also found a significant main effect of *Group* (*F*(5,149) = 5.1; *p* ≤ 0.001; *η*_*p*_^2^ = 0.15; θ↓β↑: 44.1 ± 3.7%, θ↑β↑: 32.8 ± 3.8%, θ↑: 34.8 ± 4.4%, β↑: 37.8 ± 3.8%, no NF: 51.8 ± 4.2%, TD: 25.8 ± 3.9%), indicating general group differences in performance. Most importantly, the Time*Group interaction was also significant (*F*(5,149) = 2.4; *p* = 0.04; *η*_*p*_^2^ = 0.07). We thus opted to not analyse or interpret the main effect of *Group* any further. Descriptive data are shown in Fig. 2C. To analyse this interaction more closely, we first examined group differences at the two measurement times. A significant main effect of *Group* was present both at T1 (*F*(5,149) = 4.6; *p* = 0.001; *η*_*p*_^2^ = 0.14) and at T2 (*F*(5,149) = 5.0; *p* ≤ 0.001; *η*_*p*_^2^ = 0.14).

Bonferroni-corrected post hoc tests showed significantly lower false alarm rates in the TD group compared to the no NF group (*p* = 0.001) and the θ↓β↑ group (*p* = 0.003) at T1 (Fig. 2C). At time point T2, only the patients with ADHD not taking part in NF (no NF group) still presented with more false alarms than the TD (*p* ≤ 0.001) (Fig. 2C). In all four groups treated with NF, false alarm rates did not differ from the error rate in the TD group (θ↓β↑: *p* = 0.18; θ↑β↑: *p* ≥ 0.99; θ↑: *p* ≥ 0.99; β↑: *p* ≥ 0.99). Mirroring this effect, the false alarm rate in three of the four NF groups was now significantly lower than the false alarm rate in the no NF group (θ↑β↑: *p* = 0.003; θ↑: *p* = 0.012; β↑: *p* = 0.03). Interestingly, it was particularly the θ↓β↑ group, i.e. containing patients taking part in the “traditional” NF protocol, that did not differ from the untreated (no NF) group at time point T2 (*p* = 0.58).

In addition, we examined the presence of any treatment effects (effects of *Time*) in all of the groups separately. Significant main effects of time were found in all patients with ADHD treated with either of the four NF protocols (θ↓β↑: *t*(28) = 3.4, *p* = 0.002, *d* = 0.63; θ↑β↑: *t*(27) = 2.4, *p* = 0.025, *d* = 0.45; θ↑: *t*(20) = 3.1, *p* = 0.005, *d* = 0.69; β↑: *t*(27) = 3.6, *p* = 0.001, *d* = 0.68). In contrast, no such effects were found in the no NF group (*t*(22) =  − 0.01, *p* = 0.99, *d* =  − 0.003) and the TD group (*t*(25) = 0.73, *p* = 0.47, *d* = 0.14). For descriptive data see Fig. 2C.

### Flanker Task

**Fig. 3:**
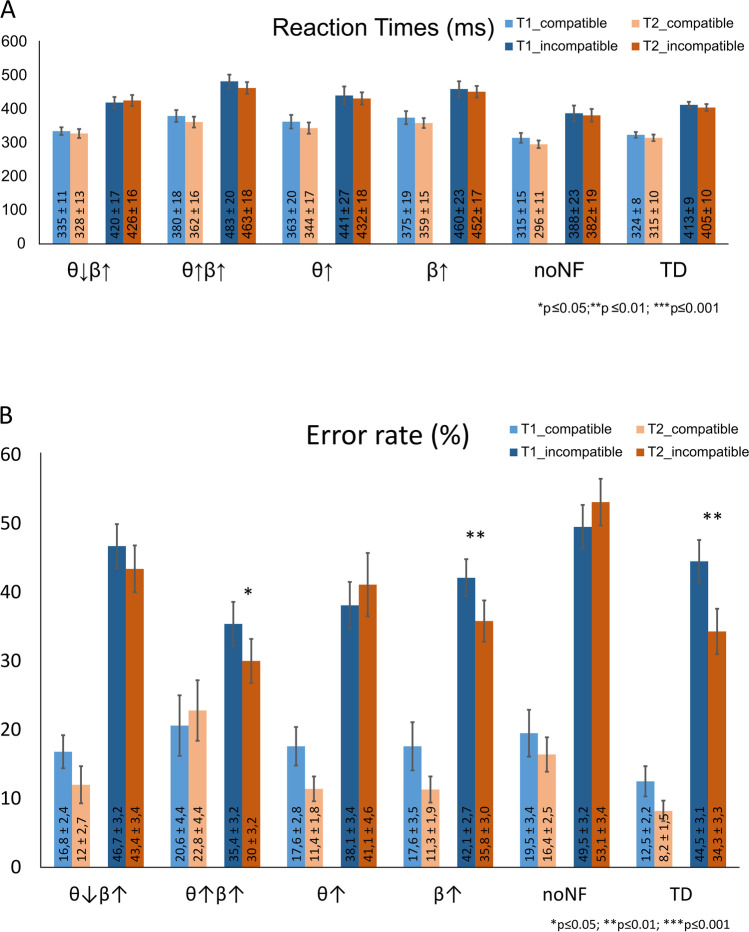
Descriptive statistics (mean ± SE) for flanker reaction times (**A**) and flanker error rates (**B**) for compatible (pale colours) and incompatible (bright colours) trials. Blue bars represent testing time point T1; orange bars show testing time point T2. Data is shown for four groups of patients with ADHD treated with four different neurofeedback protocols (θ↓β↑: trained to downregulate theta and upregulate beta power, θ↑β↑: trained to upregulate both theta and beta power, θ↑: trained to upregulate theta only, β↑: trained to upregulate beta only) and for two control groups (no NF: patients with ADHD who did not take part in any training, TD: typically developing control children). Asterisks indicate level of significance of conducted post hoc *t*-tests

Behavioural parameters showing performance in the flanker task are shown in Fig. 3.

An analysis of the reaction times (Fig. 3A) is shown in the supplemental material. Concerning error rates (Fig. 3B), we found significant main effects of *Time* (*F*(1,147) = 13.2; *p* ≤ 0.001; *η*_*p*_^2^ = 0.08; T1: 30.1 ± 1.1%; T2: 26.7 ± 1.0%) and *Compatibility* (*F*(1,147) = 427.5; *p* ≤ 0.001; *η*_*p*_^2^ = 0.74; compatible trials: 15.6 ± 1.0%; incompatible trials: 41.2 ± 1.2%). The main effect of *Group *was not significant (*F*(5,147) = 2.1; *p* = 0.07; *η*_*p*_^2^ = 0.07). Importantly, the Time*Compatibility*Group interaction was significant (*F*(5,147) = 2.3; *p* = 0.049; *η*_*p*_^2^ = 0.07). To tease the effects apart, compatible and incompatible trials were examined separately. No significant interaction of Time*Group was found within compatible trials (*F*(5,147) = 0.91; *p* = 0.48; *η*_*p*_^2^ = 0.03). Within the incompatible trials, however, this interaction was significant (*F*(5,147) = 3.3; *p* = 0.008; *η*_*p*_^2^ = 0.1). Dividing this up further, we first found significant main effects of *Group* at time point T1 (*F*(5,147) = 2.7; *p* = 0.02; *η*_*p*_^2^ = 0.08) and at time point T2 (*F*(5,147) = 5.3; *p* ≤ 0.001; *η*_*p*_^2^ = 0.15). For incompatible trials at T1, Bonferroni-corrected post hoc analyses revealed significantly lower error rates in θ↑β↑ group than in the no NF group (*p* = 0.04). After treatment (i.e. at time point T2), this difference increased even further (*p* ≤ 0.001). In addition, error rates in incompatible trials were now significantly different between the no NF and the TD group (*p* = 0.004) and β↑ group (*p* = 0.01). When analysing the effects of *Time* within each group separately (see Fig. 3B for descriptive data), the θ↑β↑ group (*t*(26) = 2.1, *p* = 0.05, *d* = 0.40), β↑ group (*t*(27) = 2.8, *p* = 0.01, *d* = 0.52) and TD group (*t*(25) = 3.1, *p* = 0.005, *d* = 0.60) revealed significant differences. This was not the case for the other two NF groups (θ↓β↑ (*t*(28) = 1.1, *p* = 0.30, *d* = 0.20), θ↑ (*t*(20) =  − 1.1, *p* = 0.29, *d* =  − 0.24)) or the no NF group (*t*(21) =  − 1.2, *p* = 0.26, *d* =  − 0.25).

### Post Hoc Power Analysis

To obtain a post hoc estimate of the robustness of our results in the Go/Nogo and the flanker task, we conducted a post hoc power analysis in addition to the a priori power analysis detailed in the “Methods” section. Our obtained effect size reached approximately *η*_*p*_^2^ = 0.1, as it was the case in previous studies (Bluschke et al., 2018, 2016a, 2016b). The outcome measures showing the relevant effects (i.e. Nogo false alarm rate and error rate in incompatible flanker trials) showed repeated measure correlations of *r* ≥ 0.63 (all *p* ≤ 0.001). Taking into consideration our sample size of *n* = 157 (6 groups, 2 measurement time points, alpha level = 5%), the power calculation using G*Power revealed an achieved power ≥ 90%.

### Parent-Rated ADHD Symptoms

Parent-rated levels of ADHD symptoms (Table 3) did not differ significantly between the five ADHD groups at T1 or T2 (all *F* ≤ 1.0, all *p* ≥ 0.44, all *η*_*p*_^2^ ≤ 0.043). All patients with ADHD demonstrated significantly stronger ADHD symptoms than children in the TD group (all *t* ≥ 5.3; all *p* ≤ 0.001). In all three core symptoms of ADHD, we found significant main effects of *Time* (all *F* ≥ 5.2, all *p* ≤ 0.025; all *η*_*p*_^2^ ≥ 0.05), indicating generally reduced symptoms at T2 compared to T1 across groups (see Table 3). Further, we found a main effect of *Group* (all *F* ≥ 7.8, all *p* ≤ 0.001; all *η*_*p*_^2^ = 0.28) concerning all three symptom domains. Regarding inattention (all *p* ≤ 0.001) and impulsivity (all *p* ≤ 0.04), Bonferroni-corrected post hoc tests demonstrated that this effect was driven by the difference in symptom levels between TD and all ADHD groups together. Hyperactivity levels, however, did not differ significantly between TD and patients included in the θ↑ group (*p* = 0.2) or in the β↑ group (*p* = 0.1). We found no significant Time*Group interactions (all *F* ≤ 1.5, all *p* ≥ 0.2; all *η*_*p*_^2^ ≤ 0.07).

### Neurofeedback Baseline and Training Data

Data are presented in Table 4.

**Table 4:** Neurofeedback training data. Neurofeedback training data (i.e. power in the theta and beta frequency at rest (baseline) and the change occurring within the first NF block of the respective training session) (mean ± SE) for all four groups treated with neurofeedback (θ↓β↑ (patients with ADHD training downregulation of theta and upregulation of beta), θ↑β↑ (patients with ADHD training upregulation of theta and upregulation of beta), θ↑ (patients with ADHD training upregulation of theta) and β↑ (patients with ADHD training upregulation of beta)). Data measured at the first and the last NF session are shown and compared using paired *t*-test. Due to technical issues, data is not available for all participants

		Theta (θ)			Beta (β)		
		First NF session	Last NF session	Paired *t*-test	First NF session	Last NF session	Paired *t*-test
θ↓β↑ values available for *n* = 18	Baseline	6.2 ± 1.2	5.9 ± 1.2	*t*(17) = 1.53; *p* = 0.14	2.9 ± 0.7	2.9 ± 0.6	*t*(17) = 0.13; *p* = 0.45
	Training	− 0.7 ± 0.1	− 0.01 ± 0.2	*t*(17) = − 0.29; *p* = 0.78	0.08 ± 0.09	− 0.07 ± 0.07	*t*(17) = 2.66; *p* = 0.017*
θ↑β↑ values available for *n* = 13	Baseline	5.9 ± 1.4	5.7 ± 1.9	*t*(12) = 0.51; *p* = 0.62	2.5 ± 0.3	2.5 ± 0.4	*t*(12) = − 0.48; *p* = 0.64
	Training	0.2 ± 0.1	0.3 ± 0.3	*t*(12) = − 0.42; *p* = 0.68	0.7 ± 0.03	0.06 ± 0.06	*t*(12) = − 0.23; *p* = 0.82
θ↑ values available for *n* = 18	Baseline	6.5 ± 1.1	6.5 ± 1.1	*t*(17) = − 0.06; *p* = 0.95	–	–	–
	Training	− 0.01 ± 0.07	0.49	*t*(17) = − 5.64; *p* ≤ 0.001*	–	–	–
β↑ values available for *n* = 24	Baseline	–	–	–	3.2 ± 0.8	3.2 ± 0.8	*t*(23) = − 0.08; *p* = 0.94
	Training	–	–	–	− 0.009 ± 0.65	0.48 ± 0.24	*t*(23) = − 2.36; *p* = 0.027*

* denotes p < .05

We examined whether there were any changes in the power (resting state) of the trained frequency bands in the four groups treated with neurofeedback. We did not observe any changes in the theta/beta frequency band power in either of the four groups (all *t* ≤ 1.53; all *p* ≥ 0.14). Further, we examined “training success” by comparing the increases/decreases achieved in power from the first to the last NF session. Here, we found a significant increase of theta power in the θ↑ group as well as a significant increase of beta power in the β↑ group. No such effects were found in the two groups training up-/downregulation of both frequency bands in parallel. In the classic NF protocol (θ↓β↑), beta power was even regulated in the opposite direction, resulting in lower beta power after completion of the NF sessions.

## Discussion

The current study compared the effects of different NF protocols modulating theta and/or beta frequency bands on two instances of cognitive control functions in children and adolescents with ADHD. It aimed to examine the effects of theoretically driven manipulations of the classical NF protocol on objective performance measures by assessing cognitive control functions which bear a significant relevance for the day-to-day functioning of patients with ADHD. We chose a Go/Nogo task measuring response inhibition and a flanker task measuring interference control. Additionally, we examined effects on parent-rated ADHD symptoms as measured by the AD(H)D Symptom Checklist as well as changes occurring in the trained neurofeedback parameters.

The results show that the “gold standard” NF protocol (i.e. θ↓β↑) only enhanced response inhibition performance and led to reductions of false alarms in the Go/Nogo task, corroborating previous results using the same task (Bluschke et al., 2018). Regarding interference control, no changes between testing time points were evident applying the standard θ↓β↑ protocol. This shows that the NF protocol being most commonly used in ADHD treatment only modulates some aspects of cognitive control (i.e. response inhibition or impulsivity), while other important aspects of cognitive control are not affected (i.e. interference control). Inhibitory control processes are enhanced by all of the tested NF protocols. Opposed to this, interference control was only enhanced when theta and beta activity was upregulated (θ↑β↑), or when only the upregulation of beta band activity was trained (β↑). Therefore, these two protocols are more powerful than the other ones in enhancing cognitive control processes in ADHD. The finding that protocols entailing a modulation of beta band activity (i.e. not the θ↑ protocol) led to most consistent effects in ADHD suggests that beta band activity is particularly important when aiming to enhance both response inhibition and interference control processes in ADHD. Theta oscillations do not seem to be as important as beta oscillations for the cognitive control processes being examined in ADHD despite several studies having shown that theta oscillations are important during response inhibition (Chmielewski et al., 2016; Mückschel et al., 2017) and interference control (Cohen, 2014; Nigbur et al., 2012), as well as in ADHD in general (Yordanova et al., 2013; Zhang et al., 2017). To explain this pattern of results, it is important to consider the possible functional roles of theta and beta oscillations in cognitive control.

There is some conceptual overlap in functions between theta and beta oscillations in terms of continuous comparison processes between a desired/expected and the actually perceived information, or the desired and the achieved response outcome. This is relevant in both tasks. Theta oscillations have been suggested to be important for cognitive control since such slow oscillation processes allow the organization of brain activity across remote areas (i.e. from basic perceptual processes to aspects of motor response execution) (Buzsáki, 2006; Cavanagh & Frank, 2014). This may particularly be the case for medial frontal theta oscillations (Cavanagh & Frank, 2014), which may reflect a “surprise signal”. Whenever expectancies, particularly of one’s own behaviour (e.g. response in a task), are not fulfilled, the continuous comparison process between expected and factual outcome is resulting in such a “surprise signal”. Thus, the theta frequency band is related to continuous comparison processes. In the sense of a sensory sampling for relevant information and the maintenance of the status quo (Engel & Fries, 2010), such comparison processes related to beta frequency oscillations are necessary when directing attention towards upcoming motor tasks (Fetz, 2013; Khanna & Carmena, 2015; MacKay, 1997; Saleh et al., 2010; Seki & Fetz, 2012). This conceptualization suggests that beta band activity is stronger when the maintenance of the status quo is likely than when a change is expected. Again, this requires a comparison process. Yet, whereas theta oscillations seem to be enhanced when there is a violation of expectancies, beta oscillations seem to be particularly pronounced when this is not the case and a current state is expected to be maintained.

In the flanker task, especially sensory sampling is more complex than in the Go/Nogo task because here (i) more complex stimuli have to be captured in less time (flanker SOA) and because there is (ii) also a choice between different responses instead of the demand to inhibit one particular response. Therefore, for the Go/Nogo task the modulation of beta band activity seems to be less crucial, possibly because demands on stimulus sampling and the closely related response selection are lower. In this context, it is important to consider that the effects of beta band upregulation are not independent of concurrent processes taking place in the theta frequency range. This is evident when comparing the effects of the standard NF protocol (i.e. θ↓β↑) to those of protocols training the upregulation of theta and beta activity (i.e. θ↑β↑) or only beta (i.e. β↑). When theta is modulated in the direction opposite to beta activity, there is no improvement in the control of interference processes as measured by the flanker task, indicating that the enhancement of beta activity is not sufficient in order to handle the more complex sensory sampling demands of the flanker compared to the Go/Nogo task. Thus, our findings demonstrate that an NF-based increase in beta band only has a consistent effect when theta activity is modulated in the same direction but not when theta and beta band activity are modulated in opposite directions. Thus, it seems that the effects of theta and beta somewhat cancel each other out when both frequency bands are modulated simultaneously in opposite directions in the NF protocol. However, this mutual cancellation of effects is most likely incomplete since positive effects of a θ↓β↑ NF protocol were still present in the Go/Nogo task with its lower demands on sensory sampling. Accordingly, an improvement in Go/Nogo performance is still possible through the application of a θ↓β↑ NF protocol. This is not the case for interference control in the flanker task which poses higher demands on sensory sampling. However, such an explanation is only plausible if theta and beta band activity are at least partially based on common overlapping neural mechanisms. Crucially, this is the case since the functional processes coded by theta and beta oscillations both relate to a continuous comparison process (see above).

This concept of different levels at which NF-related changes may or may not occur is also supported by our finding that neither of the trained neurofeedback parameters significantly de- or increased between the first and last NF session in either of the four groups when considering baseline power levels. Concerning the actual changes in the ability to up-/downregulate the required frequency band power according to the respective NF protocol (i.e. “trainings success”), we did find specific effects in the groups of participants training the upregulation of either theta or beta power. No such effects were found in the groups of participants required to train the modulation of both these frequency bands simultaneously. Overall, these findings support existing knowledge (Pscherer et al., 2019, 2020, 2022) showing that resting state power carries a different functional relevance than it is the case for task-related oscillatory power and outcomes measured on the behavioural level. This further strongly depends on age and the cognitive function in question (Pscherer et al., 2019, 2021). It is thus not surprising that the pattern of results differs depending on the analysed outcome parameter. Future research would need to examine these different coding levels and their functional relevance further. Specifically, to arrive at an in-depth understanding of how, why and when neurofeedback works, it would be relevant to also consider effects on neurophysiological measures including event-related potentials, time–frequency decompositions and source localizations. Overall, however, our results show that particularly the protocols focusing on training the modulation of one (instead of both) of the frequency bands result in increased power values at the end of the NF training. The upregulation of theta power alone was associated with measureable increases in theta power as well as improved inhibition performance. The upregulation of beta power alone, however, additionally led to improved interference control.

The obtained results differentiate previous findings by showing that beneficial effects of theta/beta ratio NF protocols on cognitive control processes are mainly due to one component of the currently used protocol, namely, the increase in beta band activity (Bluschke et al., 2016a, 2016b). Thus, previous finding showing only limited evidence for the effectiveness of neurofeedback (Catalá-López et al., 2017; Cortese et al., 2016; Razoki, 2018) might be the result of non-optimized protocols. As argued previously (Bluschke et al., 2016a, 2016b), the current practice of the θ↓β↑ NF protocol in clinical settings is based on historically routed misconceptions about the role of theta oscillations for cognition (Bluschke et al., 2016a, 2016b). In these frameworks, theta frequency activity has commonly been interpreted as an indicator of daydream-like, in-alert states, while beta band activity has been thought to reflect focused attention and mental arousal (Hammond, 2011). The current data suggest the need for a change in clinical practice and underline criticisms concerning the relative lack of a neuroscientific rationale for the application of θ↓β↑ NF protocols in clinical settings (Heinrich et al., 2014; Holtmann et al., 2014; Saad & Kohn, 2015). Previous clinical studies on ADHD and NF have focused almost exclusively on the clinical symptoms of patients and have provided some evidence for the effectiveness of a classic θ↓β↑ NF protocol in this regard (Bluschke et al., 2018, 2020, 2016a, 2016b; Enriquez-Geppert et al., 2019; Gevensleben et al., 2010, 2009a, 2009b; Lambez et al., 2020; Lofthouse et al., 2012; Riesco-Matías et al., 2021; Van Doren et al., 2019; Yan et al., 2019). In the current study, clinical data (see Table 3) revealed reduced ADHD symptoms across all groups. Since this was the case in both the NF and the control groups, it is thus unclear whether these results reflect practice or training effects. Importantly, however, improvements were comparable across all NF groups, demonstrating that neither of the applied NF protocols carries any disadvantages in terms of symptom development. Therefore, the data suggest that a change in clinical practice of frequency-based NF protocols does not only provide a benefit at the level of cognitive control processes. Rather, clinically relevant ADHD symptom dimensions are still positively affected. Nevertheless, it is important to keep in mind that all effects concerning symptom severity were main effects, indicating similar levels of improvement across all groups. Such somewhat unspecific effects of NF have been reported previously (Ros et al., 2020), demonstrating the need for further differential investigations of influential factors. Altogether, our results indicate that symptom severity and cognitive control processes represent rather distinct, maybe even independent, levels of alterations in ADHD. Further specifically designed studies are required in order to examine whether different NF-related changes on these two levels may occur due to different temporal dynamics, different patterns of variability or different plasticity patterns.

A limitation of the study is that no strictly or systematically randomized or blinded study design was used to avoid any possible systematic biases of group allocation. However, due to the naturalistic setting, group allocation took place in a quasi-random fashion with the allocation not carrying any meaning to participants. Further, assessment was blinded since experimenters conducting testings at T1 and T2 were unaware of group membership of the participants. Since our goal was to systematically analyse differential effects of variations in the frequency bands on cognitive control parameters, no qualitative rating (e.g. ADHD symptoms) took place and the study provides no conclusions concerning the effectiveness on clinical parameters. Instead, our study focused on experimental measures, for which the definition of clinical meaningfulness is difficult due to the lack of absolute or relative cut-off scores. We did not examine the precise neurophysiological processes underlying the observed behavioural changes and do not report brain activity recorded during neurofeedback. Future studies should include session-by-session data on the extent to which the desired up- or downregulation of the respective frequency bands was achieved in order to be able to derive more direct causal inferences concerning the connection between NF training and any changes in assessed cognitive control parameters, since such effects on outcomes have been demonstrated previously (Baumeister et al., 2019). This is important as it is necessary to examine the data in a more fine-grained fashion than it is possible by considering mean outcomes only (Heinrich et al., 2007). In addition, the effects of the number of conducted NF sessions also need to be examined in more detail, with the 16 sessions conducted within this study ranging on the lower end of the spectrum.

## Conclusions

In summary, including the enhancement of beta band activity in frequency band neurofeedback protocols is key when aiming to train patients with ADHD to increase cognitive control functions. These consistent enhancing effects on both response inhibition and conflict control were observed both without any concurrent theta regulation and in combination with theta upregulation. The simultaneous downregulation of theta power in addition to beta upregulation (i.e. the “traditional” NF protocol), however, resulted in much weaker effects that did not reach statistical significance. The data call for a change in the clinical usage of NF protocols and show that protocols different to the clinical standard are most effective to in enhancing important facets of cognitive control alongside clinical symptoms in ADHD.

## Supplementary Information

Below is the link to the electronic supplementary material.

Supplementary file1 (DOCX 16 KB)

## Data Availability

The datasets generated during and/or analysed during the current study are available from the corresponding author on reasonable request.

## Abbreviations

ADHD: Attention deficit/hyperactivity disorder
NF: Neurofeedback
TD: Typically developing controls

## Code Availability

The codes from the experiments used in the current study are available from the corresponding author on reasonable request.
