# Supplementary material for: The Effects of Different Theta and Beta Neurofeedback Training Protocols on Cognitive Control in ADHD
Source: J Cogn Enhanc. 2022 Nov 4;6(4):463–77. doi: 10.1007/s41465-022-00255-6 (PMC9638270; doi:10.1007/s41465-022-00255-6)
Supplement: Supplementary file 1 — Supplementary file1 (DOCX 16 KB) [file 41465_2022_255_MOESM1_ESM.docx]

**Supplemental Material**

**The effects of different theta and beta neurofeedback training protocols on cognitive control in ADHD – a naturalistic observational study**

**Results**

*Go/Nogo task*

Concerning the hits in the Go trials, we found no significant main effects of *Time* (F(1,149)=0.76; p=0.38; η_p_^2^=0.005) or *Group* (F(5,149)=1.3; p=0.26; η_p_^2^=0.04). Further, we found no significant *Time*Group* interaction (F(5,149)=0.3; p=0.93; η_p_^2^=0.009). Descriptive data for all groups are shown in Fig. 2A. Concerning the reaction times in the Go trials, we also found no significant main effects of *Time* (F(1,149)=0.4; p=0.51; η_p_^2^=0.003) or *Group* (F(5,149)=1.7; p=0.15; η_p_^2^=0.05). The *Time*Group* interaction was also not significant (F(5,149)=0.13; p=0.99; η_p_^2^=0.004). Descriptive data are shown in Fig. 2B.

*Flanker Task*

Concerning response times (RTs), we found significant main effects of *Time* (F(1,147)=4.5; p=0.04; η_p_^2^=0.03; T1: 391±7.2ms; T2: 380±6.1ms), *Compatibility* (F(1,147)=900.4; p≤0.001; η_p_^2^=0.86; compatible trials: 341±5.6ms; incompatible trials: 430±6.8ms) and *Group* (F(5,147)=3.8; p=0.003; η_p_^2^=0.12; θ↓β↑:377±13.8ms, θ↑β↑: 422±14.4ms, θ↑: 395±16.2ms, β↑: 411±14.0ms, no NF: 345±15.8ms, TD: 364±14.6ms). This demonstrates general improvements across time, the task-inherent faster reaction times in non-conflict compatible trials and general group differences in performance. Specifically, the no NF group demonstrated significantly faster reaction times than those in the θ↑β↑ group (p=0.007) or the β↑ group (p=0.033). No interaction effect was significant (all F≤3.4, all p≥0.07; all η_p_^2^≤0.02).
